# Bioelectrical Impedance Vector Analysis (BIVA) for Assessment of Hydration Status: A Comparison between Endurance and Strength University Athletes

**DOI:** 10.3390/s24186024

**Published:** 2024-09-18

**Authors:** Maria Abdelnour, Rédina Berkachy, Lara Nasreddine, Elie-Jacques Fares

**Affiliations:** 1 Department of Nutrition and Food Sciences, Faculty of Agricultural and Food Sciences, American University of Beirut, Beirut 1107 2020, Lebanon; ma709@aub.edu.lb (M.A.); ln10@aub.edu.lb (L.N.); 2 School of Engineering and Architecture of Fribourg, University of Applied Sciences of Western Switzerland, 1700 Fribourg, Switzerland; redina.berkachy@hefr.ch

**Keywords:** bioelectrical impedance vector analysis, resistance, reactance, phase angle, urine specific gravity, hydration, athletes

## Abstract

Introduction: Athletic performance is greatly impacted by hydration status. The combination of several techniques is recommended to accurately measure water losses and gains. Aim: The aim of this study is to assess the validity of bioelectrical impedance vector analysis (BIVA) as a tool for measuring hydration status in endurance and strength athletes. Methods: A total of 148 athletes were evaluated on one experimental day, pre- and post-training. Urine samples were collected and analyzed for color and specific gravity. Body weight changes were measured, sweat rate was calculated, and BIVA was performed. Reference ellipses were plotted using data of 200 healthy non-athletic individuals. Results: A moderate significant agreement was noted between raw bioelectrical values and urine specific gravity (USG) (*p* > 0.05). The sensitivity of classic BIVA in detecting minor changes in hydration status is confirmed both graphically and statistically. R/h and Z statistically significantly decreased post-training. Male athletes exhibited a specific BIA vector distribution compared to the reference population and were slightly more hydrated than female athletes. Conclusions: BIVA validation may be an essential step to allow its use among university students to assess dehydration in a non-invasive, practical, and inexpensive way.

## 1. Introduction

Water is the most essential nutrient for athletes [1]. The American College of Sports Medicine (ACSM) recommends the maintenance of adequate body hydration status before, during, and after exercise to improve training capacity and performance, decrease the risk of heat illness and injury, and to achieve euhydration. Excessive dehydration, defined as a loss of at least two percent of body weight, negatively affects performance, particularly in endurance athletes, by increasing the risk of injuries, fatigue, and perceived effort. Several factors can influence hydration status, including habitual fluid intake, exercise type, intensity and duration, sweat loss, sweat rate during exercise, and environmental conditions [2]. 

An adult’s total body mass (TBM) is made up of approximately between 58 and 65% water [3]. Body fluids can be estimated using the isotope dilution method, a safe, precise, and highly accurate method for extracellular and total fluids volume assessment. However, isotope dilution is costly and requires tedious specimen preparation [4]. Additionally, dual-energy X-ray absorptiometry (DXA) is a body composition method that provides information about TBW by measuring changes in lean body mass [4]. However, its measurements in athletes are affected by exercise and food ingestion. Furthermore, a combination of alternative methods is recommended for the assessment of hydration status to obtain accurate and valid results. Multiple methods (gross and body fluids) allow a better understanding of the location of water retention in the body in the absence of a direct assessment of intra- and extracellular hydration [3]. Other commonly used methods include urine color, urine-specific gravity, osmolality, body weight changes, sweat rate, and bioelectrical impedance (BIA) [5]. 

During exercise, or shifts in body position, changes in total body water (TBW) may vary in different body fluid compartments. TBW is not a hydration assessment method [6] since it relates to the estimation of the total volume of fluid, rather than its measurement, and it does not offer information about the distribution between intra- and extracellular spaces. Thus, it is important to follow standardized protocols to control for possible confounders when assessing hydration status. Urine color and specific gravity are two inexpensive, convenient, easy, and fast collection and analysis methods and are minimally invasive compared to blood variables for hydration assessment. Urine osmolality and urine specific gravity are considered more accurate than urine color [3]. Measurements of hemoglobin and hematocrit can be used to calculate changes in blood volume, cell volume, and plasma volume under controlled exercise conditions (temperature and posture). However, the transfer of fluid from ICW to the ECW space and renal retention mediate the recovery of serum osmolality, which lowers its sensitivity as a hypohydration indicator. Hence, urine specific gravity (USG) is a better indicator of fluid deficit. The aforementioned hydration assessment methods are considered adequate for sports science but are not practical for hydration status monitoring during training because they are expensive and necessitate methodological control and analytical expertise [7].

Changes in body mass before and after training are used to estimate the volume of sweat loss during training while correcting for fluid intake. One liter of sweat loss is estimated to be equivalent to one kilogram of body mass loss. Factors that affect sweat rate include but are not limited to factors at the individual level (genetics, the level of acclimatization, the level of fitness, and age); at the activity/sport level (intensity, intermittent vs. continuous, land vs. water, and modality); and at the environmental/external conditions level (humidity, temperature, clothing, and solar radiation).

Exercise type plays an essential role: swimmers dissipate greater body heat through convection and conduction in water compared to runners. Additionally, heat exposure (from cold to warm conditions) increases sweat rate. These factors are to be accounted for when adjusting volumes of fluid intake [2]. 

It is recommended to combine urinary measures with gross measures such as body mass, (BIA) and/or (DXA). One advantage of their use is the alleviation of the issue of using fluid from one body compartment to predict whole body hydration status.

BIA is a doubly indirect practical quantitative body composition assessment method for the estimation of TBW by passing a small electrical current through the body and measuring the resistance (impedance) to that current. It allows a quick and non-invasive assessment of TBW at 50 kHz with an error of 1.5–2.5 kg [8]. It is based on impedance and necessitates calibration against another method, deuterium oxide (D2O). Since body water conducts electricity well, the impedance measurement allows the estimation of total body water (TBW). From TBW, the volume of extracellular and intracellular fluids can also be inferred using established equations and models [4]. When using BIA to estimate body fat, it becomes a double indirect method: the primary output is an estimate of body water and the secondary output, the body water estimate, is then used to infer fat-free mass (FFM), since body water constitutes a significant portion of FFM. The proportion of body fat is subsequently derived by subtracting FFM from total body weight. This two-step process (estimating body water first, then calculating fat-free mass, and finally body fat) classifies BIA as a double indirect method when it comes to body fat assessment [9].

BIA measurements are derived from regression equations, deemed to be inaccurate in the athletic population at the individual level [5]. On the other hand, BIA presents accurate measurements for body composition and fluid volume change tracking on a group-level in athletes. BIA measurements may be affected by posture, electrolyte balance, skin temperature, food and alcohol ingestion, physical activity intensity and malnutrition and its accuracy is affected by body fluid volume changes and tonicity [4]. 

Alternatively, body composition assessment can be performed qualitatively by evaluating the bioelectrical phase angle (PA) calculated as the arctangent of Xc/R × 180°/π, where Xc is the reactance representing the delay in the flow of current measured as a phase shift and indicating dielectric properties, and R is the resistance representing the decrease in voltage indicating conductivity through ionic solutions. The phase angle is an indicator of the intra/extracellular water ratio and allows for the detection of acute dehydration when high values are recorded and acute hyperhydration or chronic dehydration when lower phase angle values are noted. In the athletic population, PA is likely higher in males than females. PA largely varies between individuals for the same sport type; however, it is unclear to what extent it differs between different types of sports. Interestingly, differences may be noted for the same sport when physical characteristics of the individual athlete differ. Also, further research is needed to understand the relation of PA to performance levels and training [10]. PA ranges between 5 and 7 degrees in healthy individuals whereas in athletes it is estimated to be 9.5 degrees where it is used as an index of muscularity [5]. 

Another method that is being used for hydration assessment in athletes is the bioelectrical impedance vector analysis (BIVA), consisting of the simultaneous evaluation of the raw parameters (R and Xc) by studying the spatial relationship between the two, known as impedance Z, which displays differences in hydration and body cell mass (BCM) and is graphically plotted as a vector for soft tissue hydration. It is a qualitative measure of soft tissue that does not depend on body size. BIVA requires the performance of multiple measurements in a short period of time for accuracy and it allows for the easy classification and ranking of athletes’ hydration, independent of body mass and equations [10,11]. Classic BIVA consists of the standardization of bioelectrical values by height H to account for inter-individual variations in conductor length (R/H and Xc/H) [12]. 

Several studies have confirmed the accuracy and replication of the BIVA technique in measuring body composition by comparing its results to DXA and the dilution technique for body hydration assessment during the competitive season in athletes [13]. Therefore, classic BIVA may detect TBW (variations mainly associated with vector length, Z) and is sensitive to the intracellular water/extracellular water (ICW/ECW) ratio. Measurements of R and Xc provide a vector of length and direction (at 50 kHz). The length of the Z vector is inversely proportional to TBW, and the PA is a measure of tissue hydration status [10,12]. However, a recent systematic review indicates that “classic BIVA” may not be reliable for the detection of dehydration in athletes on an individual level [14]. 

Furthermore, the vector position within tolerance ellipses is evaluated using BIVA, specifically drawn for each population and reflecting the percentile in body composition parameters. Reference target zones can be obtained from tolerance ellipses, useful for the identification of the individual athlete’s profile by sport type and by competitive level. To date, a limited number of papers examined raw BIA variables in athletes. Hence, there are no definite conclusions on the variation of PA between sports types (endurance vs. resistance training and recreational vs. competitive sports) [15] making it necessary to further validate BIVA for different sports types and to design reference tolerance ellipses for athletes based on sport type, age, gender, and ethnicity [14]. 

Endurance sports players have been studied more than high-intensity exercise players due to greater sweat loss during exercise [16]. Moreover, male adult athletes have greater sweat losses compared to females, despite similar fluid consumption relative to body mass in both genders [17]. Each method of hydration status assessment has its flaws, and the accuracy and validity of measures vary by situation. There is no single universally accepted method for hydration assessment in athletes. Sensitive and accurate hydration biomarkers must detect body water fluctuations of ~3% TBW [7]. Multiple measurements are necessary to increase validity and accuracy, and to reduce measurement error resulting in an incorrect categorization of hydration [4]. 

Noting the importance of hydration in sports, in the absence of sufficient data and with the need to further validate hydration assessment techniques in athletes, this study is the first of its kind to extensively explore, evaluate, and plot vector variations by gender and sport type among athletes pre- and post-training and design reference tolerance ellipses for each using classic BIVA in the Middle East and North Africa Region. This research study was undertaken to assess agreement between BIVA as a hydration monitoring tool and reference methods (urine specific gravity, urine color, and sweat rate) in Lebanese strength and endurance university athletes pre- and post-training. It also investigated the sensitivity of classic BIVA in detecting minor changes in hydration by interpreting bioelectric variables Z, R, and Xc distribution patterns by gender and sport type. Moreover, specific confidence and tolerance ellipses were designed for the Lebanese athletic and non-athletic population. 

## 2. Materials and Methods

### 2.1. Study Design and Sampling

The study included 348 individuals in total. A pre–post quasi-experimental study was carried out between September and November 2023, among 148 AUB endurance and strength athletes. The sample size was calculated based on a 95% confidence level, 5% margin of error, 0.8 statistical power, and 50% response rate. 

#### 2.1.1. Athletic Population

Male athletes *(n =* 90) were divided into (*n* = 54) strength and (*n =* 36) endurance and female athletes (*n =* 58) were divided into *(n =* 29) strength and (*n =* 29) endurance, respectively [18]. They were selected by convenience from the American University of Beirut’s varsity teams and the Charles Hostler Student Center (CHSC) gym, respectively. 

#### 2.1.2. Reference Population

The study also included 200 non-athletic individuals equally divided between males *(n =* 100) and females (*n =* 100) constituting the reference population, selected by convenience from CHDC database, to plot reference ellipses for the athletic population.

#### 2.1.3. Selection Criteria

To be included in the study, participants must be healthy adults between the ages of 18 and 35 years, part of the American University of Beirut (AUB)’s varsity team or weightlifter members at the Charles Hostler Student Center (CHSC), not injured at the time of the study, training at least 3 times per week, females in a post-menstruation state with the ovarian cycle between days 5 to 11, not on contraceptives or drugs for menstrual cycle treatment, and not pregnant or planning to become pregnant. 

Individuals were excluded if they were not part of AUB’s varsity teams, not weightlifters at CHSC, not able to visit the clinic facility, injured/having health problems/previous surgeries that alter body composition (bariatric surgery), pregnant, or on contraceptives.

### 2.2. Recruitment

Participants were recruited by the investigating graduate student by direct approach to take part in this study. Individuals were approached while exercising at the gym and during training hours for varsity teams, in the afternoon depending on each team’s training schedule. The student investigator approached team members before their training session and informed them about the study. Also, advertisements were used to recruit athletes and they were contacted through WhatsApp and email. Flyers were posted at CHSC gym, in training rooms of varsity teams and in different locations on campus. Eligibility for participation in the study was determined by assessing if the individual meets the inclusion criteria through answering a series of questions posed by the student investigator in Charles Hostler Diet Clinic (CHDC) after obtaining consent, using the “eligibility questionnaire” (refer to Appendix C). 

Participants who voluntarily consented to participate in the study were informed that they would be asked to present one week before the experiment and one day before the experiment, and they were sent the exact time and day on which they were required to present to the CHDC through email invitations discussing the necessary steps prior to the experiment. All arrangements and experimental procedures were performed by the student investigator.

### 2.3. Data Collection

**Figure 1:**
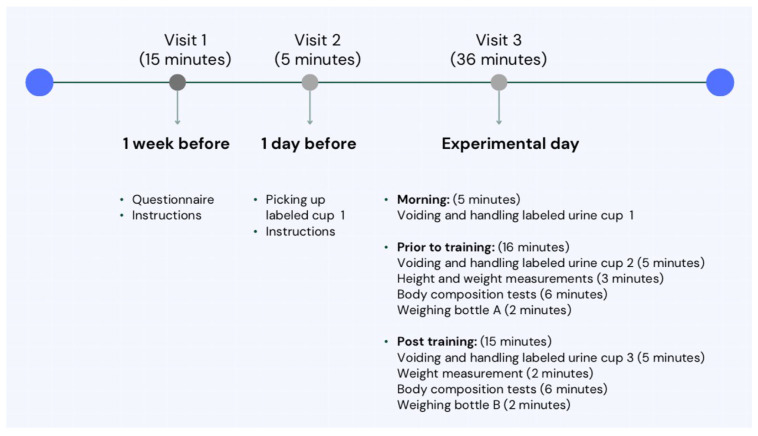
Summary of the experimental procedure.

The study took place during the fall semester at CHDC located on the first floor of CHSC at the American University of Beirut. Measurements were taken on one experimental day, during two experimental sessions, one prior to training and the second after training, to assess the hydration status of athletes. (Figure 1).

#### 2.3.1. Pre-Hydration Assessment Questionnaire

One week prior to the experiment, a short questionnaire was administered to participants (Refer to Appendix D) to gather information regarding their lifestyle and to provide them with the necessary recommendations to maximize their readiness for the study. It included questions about demographics, medical history, physical activity level, type of sports and frequency, diet, and anthropometry. The questionnaire provided the researcher with additional insight about the individual’s medical, nutritional, and health status, making it easier to select individuals based on the inclusion criteria. Furthermore, the following instructions were discussed to reach euhydration prior to the experiment: the avoidance of caffeine and alcohol consumption 4 h prior to the experiment, not exercising intensely 12 h prior to the experiment, and fasting 4 h prior to the experiment [18]. 

This questionnaire helped in recruiting participants based on the inclusion/exclusion criteria and provided the researcher with information about the medical, nutritional, and health status of participants prior to the experiment to familiarize them with testing protocols and instructions. 

On the day of the experiment, a data collection sheet was used (refer to Appendix E) to record the athlete’s first name, middle name, last name, date, time, email address, participant #, phone number (optional), and the total training duration. Additional information that was recorded included the athlete’s gender (male/female), date of birth, sport type, whether the eligibility questionnaire and pre-hydration assessment questionnaires were filled out, the empty urine cups’ weight, the time of urine collections, height, body weight measurements, water bottle weight before (A) and after (B) training and whether all 4 BIA tests were performed (2 prior to training and 2 post-training).

#### 2.3.2. Anthropometric Measurements and Body Composition

Height was measured using a stadiometer (portable, SECA 213, Hamburg, Germany). Measurements were taken in an orthostatic position and barefoot on a platform [19]. 

The body composition test was performed to obtain bioelectrical data including resistance (R), R/h (where h is the height of the individual), reactance (Xc), Xc/h, phase angle (PA), impedance (Z), and extracellular water/total body water ratio (ECW/TBW) using the InBody 770 machine (Biospace, Los Angeles, CA, USA), at a 50-kHz single frequency [20]. BIA analysis was performed four times for athletes: twice prior to training and twice post-training for accurate results. For the reference population, bioelectrical data were obtained once for each individual. Moreover, participants were also asked to remove any metals, earrings, or rings during measurements. 

The following steps were followed for the BIA test: First, participants were asked to remove shoes, socks, heavy clothes, and items in pockets. Then, they were asked to wipe their hands and feet with an electrolyte tissue provided by InBody.

Second, they were asked to stand on the device barefoot and align their heels with the round silver electrodes and the rest of the feet with the foot electrode while staying still and waiting for weight to be measured. 

Third, after weight was measured, the student investigator entered the age, height, gender, and a unique ID for participants to track the difference in measurements before and after training. 

Fourth, participants were then asked to grab the hand electrodes by placing their thumbs on the thumb electrodes and wrapping their fingers around the bottom electrodes, keeping their arms relaxed and extended slightly away from the torso so that their armpits were not touching one another (around 15 degrees). 

The BIA test took 60 s and results were automatically shown on the connected PC screen. Results were saved in a folder which allowed for the comparison of measurements before and after training. Confidence and tolerance ellipses were then created based on BIVA values [19]. 

#### 2.3.3. Urine Collection

Urine cups labeled 1 were provided to athletes at the CHDC one day prior to the experiment. Athletes were instructed on proper midstream voiding and the student investigator filled in the information regarding the date and time on the data collection sheet [21]. They were instructed to void in the morning in cup 1 and to hand it back as soon as they arrived at the university. Urine cups were weighed empty prior to hand to athletes and the empty weight was saved as a reference. Cups were labeled using numbers and letters; for example, the first morning cup was labeled as B001_MOHYAS (the first 3 letters from the first and last name of the individual) so that the individuals’ cups would not be mixed up prior to analysis. Once the urine inside each cup was analyzed, the label was discarded and cups were emptied and were only accessed by the student investigator. 

Moreover, on the day of the experiment, each athlete was given another urine cup labeled 2 after returning the cup labeled 1, and they were asked to void in the cup labeled 2 right before training. Right after the training session, they were asked to void in a cup labeled 3 and give it back immediately.

After collection, samples were analyzed in the Agr 517 Lab, Faculty of Agriculture and Food Sciences, AUB, for color scoring and urine specific gravity measurements. 

The seven-level urine color chart was used to classify individuals as hydrated (1), well hydrated (2), very well hydrated (3), extremely hydrated (4), mildly dehydrated (5), dehydrated (6), or severely dehydrated (7) [22] (refer to Appendix A). 

A urine specific gravity refractometer (Pen–Urine S.G., Atago, Tokyo, Japan) was used to measure urine specific gravity. Urine specific gravity values were categorized based on the National Athletic Trainers’ Association position statement [6] (refer to Appendix B).

#### 2.3.4. Sweat Rate

Sweat rate was assessed by calculating body mass changes. Pre-exercise body mass was measured after voiding before the start time of training, and fluid intake was tracked after this measurement. Post-exercise body mass was taken after voiding and the following equation was used [23]:

Whole body sweating rate (WBSR) = [Body massPre-Ex − (Body massPost-Ex − fluid intakeEX)]/exercise duration

Body weight was measured individually before and after training using the BIA machine right after voiding in the urine cup. returning. They were asked to remove their shoes and heavy clothes, stand with their feet on the center of the scale, and remove accessories. For the measurement of post-training weight, the same conditions were applied, after voiding in the third urine cup and without sweaty clothes.

Moreover, sweat rate analysis necessitates the collection of the volume of fluid consumed (in liters) during training, therefore, each athlete’s water bottle was weighed before and after training and the weight difference was recorded using a calibrated scale. 

Furthermore, the total training duration in hours was obtained from the athlete and training schedules.

### 2.4. Ethical Considerations

Participation in this study was voluntary, and the individual had the right to terminate his participation at any point during the experiment. 

Individuals who agreed to participate signed the informed consent provided by the student investigator at the CHDC and approved by the American University of Beirut’s Institutional Review Board (IRB), BIO-2023-0121. The consent form included a brief description of the study and highlighted what would be required from the participants and the time frame of the study. All rights and privacy are preserved by signing the consent form. 

Also, participants’ privacy is protected by keeping the responses in this study confidential, storing the data in a password-protected computer, and presenting research report findings on a group basis without any personally identifying information. Participants were informed about the protection of their privacy in the consent form.

The data are stored as coded data on a password-protected computer that only researchers have access to and will be destroyed 3 years later.

The privacy of participants was maintained by only having the participant and student investigator present in the room at the time of asking questions and noting their answers. 

### 2.5. Statistical Analysis

To assess hydration status, classic BIVA was used and compared to the following methods: urine color, urine specific gravity, sweat rate, and phase angle. R statistical software (version 4.2.3, R Foundation for Statistical Computing) was used to generate Bland– Altman plots and boxplots and to perform linear regression (refer to Supplementary Material Figures S2–S4). Canonical correlation analysis (CCA) was performed to emphasize the multidimensional multivariate aspect of the data, and the potential relation between BIVA variables and each of urine and sweat rate variables. Statistical Package for the Social Sciences (SPSS, version 25) was used for the Spearman correlation test for urine color and raw bioelectrical parameters. Descriptive statistics were also generated for urine specific gravity. Multivariate repeated measures ANOVA was performed for USG morning, USG PRE, and USG POST between the 4 sports groups. A one-sample paired Hotelling’s T-test was used to analyze pre-to-post training changes in the vector through the 95% confidence ellipses. A two-sample Hotelling’s T-test was used to determine the BIA vector differences between sports groups and the reference population by studying multivariate raw BIA data. Welch’s two-sample T-test was used to compare univariate BIA data between sports groups and reference groups. *p* < 0.05 was considered significant. Point graphs and R-Xc mean graphs were performed using the BIVA 2002^®^ software [24]. All bioelectrical impedance raw values were standardized by height (meters).

## 3. Results

### 3.1. Urine Color and BIVA Parameters

The strength of the relationship between urine color and each of the Xc, R, and Z raw values is examined using Spearman’s rho correlation in Table 1 shown below. None of the raw values were significantly correlated with urine color because these correlations are low (<0.75) Furthermore, boxplots showed no significant association between the two variables (refer to Supplementary Figures S1–S4).

### 3.2. Agreement between BIVA and (USG and SR)

#### 3.2.1. Bland–Altman Plots

**Figure 2:**
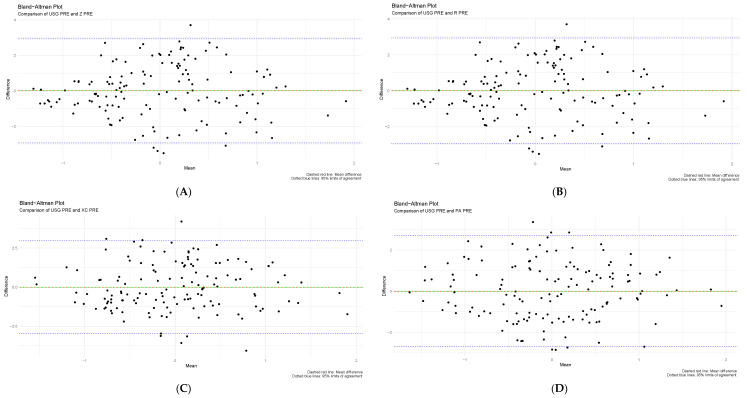
Comparison of urine specific gravity before training (USG PRE), and bioelectrical impedance vector analysis values using Bland–Altman plots. (**A**) USG and Z, (**B**) USG and R, (**C**) USG and Xc, and (**D**) USG and PA.

**Figure 3:**
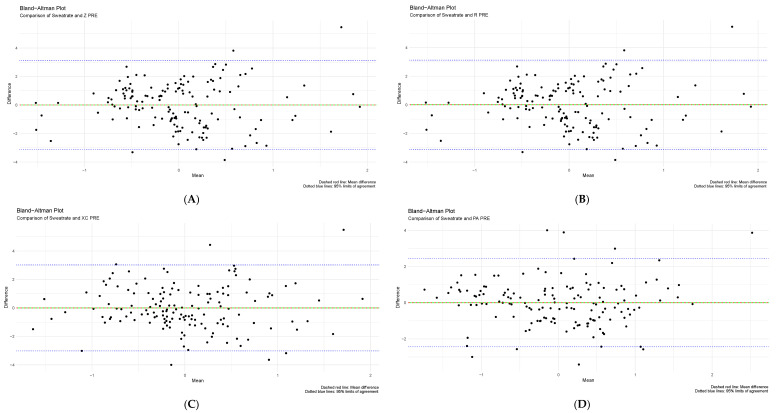
Comparison of sweat rate (SR) and bioelectrical impedance vector analysis values using Bland–Altman plots. (**A**) SR and Z, (**B**) SR and R, (**C**) SR and Xc, and (**D**) SR and PA.

A Bland–Altman plot was used for an exploratory assessment of agreement between BIVA and reference hydration assessment methods (urine specific gravity and sweat rate). The difference between measurements of two standardized datasets was calculated, followed by a one-sample independent T-test. Pre- and post-training values of USG and sweat rate were compared with raw bioelectrical impedance raw values Z, Xc, and R standardized for height, respectively. All T-tests had a *p*-value of one, strongly indicating no statistical difference between the two compared measurements. Moreover, the majority of data points fell between the 95% upper and lower limits of agreement indicating a high level of agreement between techniques. The mean difference of zero is shown in the plots below, suggesting no systematic bias. No proportional bias is noted since all plots have approximately equal numbers of data points above and below the mean difference line. Results are further explained in Figure 2 and Figure 3 below.

#### 3.2.2. Canonical Correlation Analysis

A multivariate multidimensional study of the variables was performed through the canonical correlation analysis, showing the multifaceted aspect of groups (Table 2).

Four canonical dimensions have been constructed for each dimension, by maximizing the correlation between the two sets of variables. The number of dimensions was chosen as the minimum of the number of variables in the groups BIVA and other methods.

A moderate significant correlation of 0.4124 was obtained between BIVA and other methods. Raw canonical coefficients and loadings were then calculated to explore the contribution of each variable to the canonical dimensions, and to assess the significant role of variables in the relationship between groups, respectively. These were followed by statistical tests on the canonical dimensions to understand whether combinations between BIVA variables and USG and SR variables are significant. 

The raw canonical coefficients are interpreted similar to regression coefficients. For the variable Z, a one unit increase in Z leads to a 5.7831757 decrease in the first canonical variate of the BIVA set when all the other variables are held constant. 

Moreover, the variable USG has a big influence on the four dimensions. For instance, a one unit increase in USG leads to a 36.3274513 decrease in the first canonical variate of the other methods set when all of the other predictors are held constant. 

In the BIVA set, PA, R, and Z are the ones playing important roles for dimension 1. On the other hand, for dimensions 2 and 4, Xc plays an important role.

The highest cross-loading is for sweat rate (−0.310), indicating a moderate inverse relationship between this variable and the first canonical dimensions of the BIVA dimensions. This suggests that as sweat rate increases, the first BIVA dimension decreases.

The highest cross-loadings are for R (0.389) and Z (0.389). This suggests a moderate cross-loading positive relationship between these BIVA variables and the first canonical dimension of the urine group. This implies that changes in resistance R and impedance Z are well associated with the first urine canonical dimension. They share then some common variance. PA shows a moderate negative cross-loading (−0.371) with the first urine canonical dimension, indicating an inverse relationship.

In the other methods set, sweat rate and urine color are the ones playing important roles for dimension 1, whereas for dimension 2, all three, urine color, USG, and body mass loss, are playing a significant role. For dimension 3, urine color, body mass loss, and sweat rate, are playing a significant role, and for dimension 4, USG is playing the greatest role.

The first set of canonical variates (one to four) is statistically significant, as indicated by the significant Hotelling–Lawley Trace with a *p*-value of 0.0082. This suggests that the first canonical variate explains a meaningful relationship between the two sets of variables. This is confirmed by Roy’s Largest Root test, which strongly supports the significance of the first canonical dimension in the analysis. This means that the first canonical correlation (dimension 1) captures a significant relationship between the two sets of variables. This confirms that the first canonical dimension/relation is not just statistically significant, but it is the most important. This reinforces the conclusion that most of the meaningful relationship between the two groups of variables is captured by the first canonical dimension.

Based on this analysis, one can conclude that there is a moderate significant relationship between the BIVA variables and USG: one moderately induces the other one. Although the statistics do not indicate a very strong relationship, these show a noticeable and meaningful one, in addition to being statistically significant. The moderate level could be explained by the extreme cases in the data set that might be influencing these results. 

This further confirms the potential agreement between BIVA variables and USG, which was obtained through Bland–Altman exploratory analysis. 

#### 3.2.3. Examination of Data Characteristics and Comparative Analysis

The null hypothesis of this test is that there is no statistically significant differences between the means of USG morning, USG PRE, and USG post between the four sports groups. A *p*-value of 0.110 indicates that there is not sufficient evidence to reject the null hypothesis at a significance level (e.g., 0.05). There is no statistically significant difference in urine specific gravity values among the conditions (pre-training, post-training, and morning). The partial eta squared value of 0.129 indicates a moderate effect size. (Table 3 and Table 4).

### 3.3. Pre–Post Measurements

In Table 5, the delta value for the difference between post–pre for all variables indicates minimal changes if not nonnegligible. The delta value/h standardized by height is similar to the delta value. Moreover, the paired *t*-test performed between pre and post variables has corresponding *p*-values indicating a statistically significant difference observed for all raw bioelectrical variables R/H, Xc/H, Z, and PA in the athletic population as a whole. In the male group, a statistically significant difference was noted for all, with a *p*-value of less than 0.05, except for PA with a *p*-value of 0.07, indicating it is the weakest among other variables. In the female group, R/H, Xc/H, and Z were statistically significantly different post-training whereas PA was not, with a *p*-value of 0.27.

In strength athletes, only R/H and Z were statistically significantly different. In endurance athletes, all were statistically significant, except PA with a *p*-value of 0.11. 

Cohen’s d values, which measure the effect size, are shown in the seventh column, indicating a negligible difference between pre and post PA measurements in males, Xc/h and PA in females, PA in strength athletes and Xc/H in endurance athletes, with a Cohen’s value of 0.2 or less, indicating a small effect size. Hence, the statistically non-significant difference in these groups is confirmed.

A vector displacement is noted to the left in male athletes, with a decrease in R/H and short vector lengths. Most individuals are located outside the tolerance ellipses with a shift downwards to the right on the graph. Endurance females are more deviated from the reference population compared to males, with most of them locating in the 75% tolerance ellipses. The vector position in females is slightly shifted to the left, on the minor axis (Xc) of tolerance ellipses. Minor changes are noted post-training, with less endurance female athletes located in the 50% and some shifted to the 95% tolerance ellipses. Strength females and males are mostly located in the 95% tolerance ellipses. No significant mean vector displacement is noted in any group pre–post training.

The vector of the mean difference is located near the origin of the RXc graph for both endurance and strength male athletic groups and is slightly shifted to the right. The mean impedance vectors of female athletes have overlapping 95% confidence ellipses.

Table 6 shows the obtained test statistic and *p*-value from Hotelling’s *T*-test to compare hydration status differences using BIVA in a multivariate space. The assumptions of this test are tested and considered as met. The considered model is Xc/H pre + R/H pre + Z/H pre + PA pre ~ Group (X or Y). The tested null hypothesis is that there is no statistically significant difference between the means of the athletic and reference population. The tested alternative hypothesis is that there is a statistically significant difference between the means of the athletic and reference population.

A statistically significant difference in the mean BIA vector of the athletic population and male athletic population in comparison to the reference population is noted (*p*-value < 0.05 noted by ***). The female athletic population’s mean BIA vector distribution is not statistically significantly different than that of the reference population (*p*-value = 0.3208 ⇔ 0.05) Both strength and endurance athletes have a different mean vector distribution than the general population, and this is stronger for the strength population (*p* = 0.000179). Additionally, no statistically significant difference is noted in the vector shift from pre- to post-training in the same group comparison for endurance females, strength females, endurance males, and strength males (*p*-value > 0.05).

Table 7 shows the obtained test statistic (T), degree of freedom (df), *p*-value (*p*), 95% confidence interval (CI), and means $\bar{x}$ and $\bar{y}$ of the sports and reference groups, respectively. Welch’s two-sample *T*-test was used to compare hydration status differences using BIVA in a multivariate space between two different populations. The assumptions of this test are tested and considered as met. Each of the raw BIA variables Xc/H pre, R/H pre, Z/H pre, and PA pre were tested individually between groups. 

The tested null hypothesis is that the true difference in means is equal to zero between the sports group and the reference group of each column. The tested alternative hypothesis is that the true difference in means is not equal to zero between the sports group and the reference group of each column. 

Z/H is statistically significantly different between all studied groups. R/H and PA are only statistically significantly different between male endurance athletes and male reference athletes, and between male strength athletes and male reference athletes, respectively, with *p*-values < 0.05 ***. Xc/H is only statistically significantly different between strength males and references males (*p* = 0.01703).

## 4. Discussion

This study is the first to explore raw bioelectrical values between strength and endurance university athletes from both genders and to plot reference and tolerance ellipses for the general Lebanese population. It is also the first of its kind to compare raw bioelectrical impedance vector variables to USG, sweat rate, and urine color. 

Reproducibility of the BIA device is confirmed in Table 8. Nevertheless, no significant relationships were found with urine color in the present study (refer to Supplementary Material boxplot Figures S1–S4). Urine color on its own has been deemed to be a poor hydration assessment tool, and in the present study, Spearman correlation proved the same, which could explain the absent correlation with raw BIVA values (Table 1). Urinary measures do not represent real-time hydration status at the cellular level, but rather the renal response to fluid homeostasis [4]. Urinary markers, including color and osmolality, did not correlate well with hydration status post-training [16] despite participants arriving in a euhydration state. Therefore, no further testing for agreement was done. The very similar median values for raw bioelectrical variables between dehydrated and hydrated groups according to urine color could further explain the inaccuracy of urine color. Also, these results can be altered by the diet and/or vitamin intake. On the other hand, the agreement between raw bioelectrical values standardized for height, Xc/h, R/h and Z, and PA, with each of USG and sweat rate makes all three techniques interchangeable (Table 2).

Moreover, the distribution pattern of raw bioelectrical values before and after training did not significantly change in any of the four studied groups of athletes due to the short training duration not exceeding 1.41 h on average, including rest (Table 9 and Table 10). On the other hand, the lack of detection of hydration changes post-training using other variables could be affected by the well hydrated status of individuals who were aware of the study design and were biased to drink enough water between trainings. However, the amount of water intake at the end of the training should not have affected the variables since research indicates that recent ingestion of a beverage, less than one hour from the ingestion to the next BIA measurement, seems to be “electrically silent” and to have a negligible effect on whole-body impedance Z [25]. No significant differences in USG were noted when comparing values of morning, before, and after training (Table 3 and Table 4). Thus, the agreement between USG and raw BIA variables in terms of no sensitive detection of hydration changes following training is confirmed. Hence, not only Bland–Altman plots confirm the agreement between the two methods, but also the pre–post analysis using multivariate analysis and CCA. Fernandez-Elias et al. [14] highlights urine specific gravity (USG) as a valid and practical tool for assessing hydration status in athletes, offering valuable insights into fluid balance and hydration levels crucial for optimizing athletic performance.

Only R/h and Z statistically significantly decreased post-training in both genders and sports types, to a small extent, indicating their significant role as detectors of changes in hydration in this study. Similar results were observed in a cross-sectional study [26] whereby a decrease in the R/h was noted with no change in Xc/h in elite soccer players. Resistance may be affected by temperature control, whereby every 1.0 °C increase in the skin can result in around an 11% decrease in R [27]. Additionally, vector migration along the major axis due to increased R/h and Xc/h indicates fluid loss in swimmers [20]. Hence, in the present study, the significantly decreased resistance experienced by the athletes could be reflecting the increase in body fluids. The statistically significant decrease in Z post-exercise could indicate a decrease in TBW [28]. 

**Figure 4:**
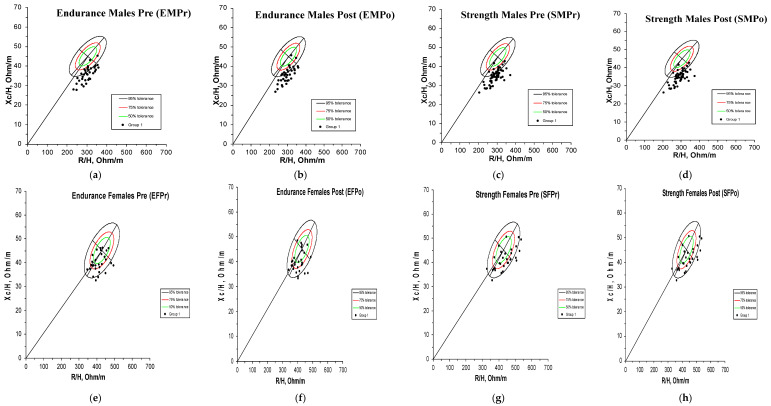
Point graphs of individual and mean impedance vectors of athletes before and after training plotted on the 50%, 75%, and 95% tolerance ellipses of the healthy Lebanese reference populations. (**a**) Endurance male athletes before training, (**b**) endurance male athletes after training, (**c**) strength male athletes before training, (**d**) strength male athletes after training, (**e**) endurance female athletes before training, (**f**) endurance female athletes after training, (**g**) strength female athletes before training, and (**h**) strength female athletes after training.

Similar to the study conducted in swimmers, a displacement to the left was also observed, due to a decrease in the R/h component (Figure 4). Other studies have also reported a shift in vectors of athletes to the left when compared to the reference population [26,29,30], which might be reflecting the specific adaptations of body composition in different sports [31]. 

It is essential to explore whether the shift in vectors is due to performance level [29] or is affected by the level of exercise [26]. 

Other studies that detected and interpreted changes in reactance after training, such as increased Xc, explained it by a possible fluid shift between intra- and extracellular compartments [30]. Additionally, PA was the weakest parameter in terms of significant changes post-training. PA has not been proven to be a reliable parameter for hydration comparison between different sports types in athletes, making it only useful for within-athlete monitoring of changes in body composition until more research is conducted [32]. Several factors can affect PA measurements including exercise level and age, necessitating further exploration [15]. 

Furthermore, male athletes were more hydrated in comparison to the reference healthy non-athletic population and compared to females, with a decrease in R/H, and they were located outside the tolerance ellipses with a shift downwards to the right on the graph. 

Endurance females were more deviated from the female reference population, in contrast to male athletes, with most of them located in the 75% tolerance ellipses. The vector position in females is slightly shifted to the left, on the minor axis (Xc) of tolerance ellipses, indicating higher BCM. Similar to males, post-training, minor changes were noted in the graphs, with fewer endurance female athletes located in the 50% and some shifted to the 95% tolerance ellipses. This could be an important indicator of the sensitivity of the BIVA technique in detecting minor changes in hydration status visually shown on graphs, giving it the advantage over other techniques including USG and sweat rate in being more precise as to the subject displacement on the graph. 

As for strength females, they were mostly located in the 95% tolerance ellipse prior to training and remained in the same position post-training. This is in accordance with the lack of change in graph visualization for strength males from pre- to post-training. Hence, graphs give us insight on the minor changes in hydration status in endurance females and endurance male athletes following training. Endurance sports athletes are more prone to dehydration post-training compared to strength athletes, regardless of gender, due to higher sweat rates and fluid losses incurred during prolonged aerobic exercise [33]. 

Moreover, graphs indicate that all sports groups are adequately hydrated; however, slightly better hydration status is noted in male athletes compared to female athletes. Similarly, the evaluation of fat-free mass hydration showed that male athletes had a mean TBW of 43.3 Kg and female athletes had a mean TBW of 31.9 Kg [34]. This could be due to males drinking greater amounts of water between the first and second BIA measurements. However, more outliers were noted in females compared to males which could have affected the results.

Findings from R-Xc mean graphs confirm those of USG, indicating no major changes in hydration status post-training, giving one more reason for validating BIVA as sensitive in detecting minor changes. 

**Figure 5:**
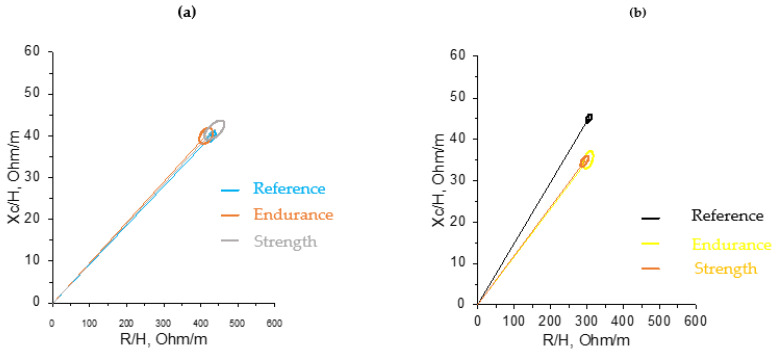
R-Xc mean graph showing the 95% confidence ellipses for the mean impedance vectors of endurance and strength athletes before training and the healthy reference populations. (**a**) Females before training and (**b**) males before training.

The overlapping 95% confidence ellipses for the mean impedance vectors of female athletes, divided into endurance and strength sports in both cases before and after training, indicate that no group is significantly more hydrated than the other (Figure 5). In other terms, sports type was not a factor that affected hydration status in females. Both endurance and strength females are very close to the reference population as shown on the graph and this is in agreement with the Hotelling’s *T*-test between the athletic and reference female groups (*p*-value = 0.3208) indicating similar hydration characteristics for both. This may imply similarities in body composition between the two female endurance and strength groups. Hence, again, graphical and statistical results are in agreement, indicating the accuracy of results in the present study. In the same context, fat-free mass (FFM), skeletal muscle mass (SMM), TBW, and ECW composition were found to be lower in endurance females compared to team sports and can be influenced by the design of the training program and requirements of each to maximize performance [35]. 

Male athletes exhibit a specific BIA vector distribution in comparison with the healthy reference population that is non-athletic and of similar age, in contrast to females. Also, no mean vector displacement from pre- to post-training was observed and this is confirmed by Hotelling *T*-tests. 

A shorter vector length was observed for males on the graph compared to females. The length of the vector is inversely related to TBW [20]. This can be explained by the consumption of water due to heat and sweat loss, impacting the BIA results. Moreover, vector length could be a key indicator of hydration status worth exploring [36]. In the same context, according to Welch’s T-test results, all four raw BIA variables (Z/h, R/h, PA, Xc/h) being statistically significantly different between strength males and reference males confirms the different distribution between the mean BIA vector of strength males and reference males. In general, factors such as training, large muscle mass, and greater glycogen reserves in athletes affect total body fluid, and hence have been shown to result in an increase in soft tissue mass in comparison to the reference population in other studies [31], indicating more water transport to the muscle [20]. Hence, more water is transported to the muscle [37]. One possible confounder could be that the reference population subjects have a great muscle mass that they had built through regular training during their life in the past years even if in the present they do not practice sports, and this could possibly be due to the greater genetic inheritability of the body composition in men compared to women [38]. 

The vector of the mean difference is located near the origin of the RXc graph for both endurance and strength male athletic groups and is slightly shifted to the right, indicating a very slight decrease in water and cellularity post-training. Both the slight decrease in BCM noted by the BIA vector and the vector differences due to decreased R/h with similar Xc/h values could be an indication of different intracellular water (ICW) content. On the other hand, the change in vector is a function of extracellular water (ECW) changes [39], in the absence of the penetration of the 50 kHz current to cells [40]. One can conclude that BIVA would allow a further understanding of ECW fluctuations and ICW content together, noting that females, for example, exhibit different body water composition in their follicular phase compared to the luteal phase of their menstrual cycle. 

Although no difference is detected using Hotelling’s *T*-test for mean BIA vector for female athletes and the female reference population and it is confirmed on the graph, a small difference is detected between the two female sport groups themselves (*p*-value = 0.77 for endurance and *p*-value = 0.64 for strength). On the graph, the ellipses are overlapping and this necessitates further exploration using Cohen’s as the small sample size could impact the significant difference. On the other hand, the two-sample Welch’s *T*-test which only indicates differences in Z/H in the univariate model analysis between each female sport group and the reference female group explains the overlapping of mean vectors on graphs. Also, graphs of males clearly illustrate results obtained through Hotelling’s *T*-test. Of relevance is the smaller female sample size (*n =* 29) for each of the endurance and strength sports in comparison to the larger sample size of strength males (*n =* 54) and endurance males (*n =* 36), possibly impacting results and resulting in discrepancies between the groups.

Furthermore, other studies compared BIVA to body mass changes [20,41]; however, in the present study, the focus was on USG since it is a strong predictor of hydration status. On the other hand, sweat rates may widely vary inter-individually due to the influence of factors such as the gender of athlete, physical activity patterns, and environmental conditions [42]. 

BIVA can be a promising technique to assess hydration variations in real time and substitute hydration biomarkers that require a mobile laboratory and its use as a complementary measure to other hydration indicators will allow the parameterization of its values [20]. 

In contrast to other findings that indicated an association between athletic body composition and hydration changes [20,26,29,30,43,44], the present study did not find any impact of body composition on hydration changes post-training, mainly affected by the short training duration. Therefore, significant changes in body composition require prolonged and consistent training regimens with adequate intensity and volume [45]. 

Only a few studies have applied BIVA to evaluate short-term vector changes from pre- to post-training. An increase in R and Xc was reported after exercise along with a vector migration, indicating a decrease in body fluid because R is the opposition of the conductor to the flow of current [20,30,36]. Xc is interpreted to detect fluid shifts between intra- and extracellular compartments [30] and higher Xc and PA values have been related to lower ECW and higher ICW content [46]. Hence, further large-scale controlled studies are needed to understand Xc changes post-exercise. 

### 4.1. Strengths

To our knowledge, this is the first study that investigated agreement between BIVA technique for hydration assessment in athletes and three other commonly used methods (urine specific gravity, sweat rate, and urine color). It is also the first study to use BIVA in Lebanon to assess the hydration status of athletes and design reference tolerance and confidence ellipses for the adult Lebanese healthy population that can be used in future studies. No other studies had established tolerance ellipses for Lebanese athletes to allow for a more enriching analysis. The large sample size and the repeated BIA and USG measurements allowed for greater precision, statistical power, and accuracy. Another strength of the study is the comparison between male and female endurance and strength athletes, which has not been explored before. Also, reminders and tips from the researcher ahead of the experiment made the process smooth and served well for preparing participants.

Data were collected and analyzed by the same researcher throughout the study which maintained consistency across the results.

### 4.2. Limitations

The study has some limitations including the quasi-experimental design with no blinding of the procedure. This could have biased athletes to drink more water before and during the experiment. Moreover, some participants fasted for 4 h whereas others fasted for 8 h and this could have affected the accuracy of BIA measurements and group analysis. Additionally, some individuals trained in the morning while others trained in the afternoon; hence, those performing the experiment in the morning voided and had their BIA measured in a fasted state, whereas those training in the afternoon consumed their last meal 4 h prior to the experiment. The ingestion of food may have influenced Z to a small extent. On the other hand, some participants had to drink a lot of water to void even if they were not thirsty. Lifestyle habits such as sleep pattern, eating, smoking, drinking habits, temperature, season, humidity, and varying training durations among strength athletes could have impacted the results and were not controlled for. Furthermore, some athletes sweat heavily while others sweat less. Moreover, those who are regular consumers of creatine could have increased water retention in muscles. Additionally, patients reported orally whether they were taking any medications or if they had any health problems; some had never had a blood test. Moreover, the female sample size is relatively smaller than the male sample size due to the low number of female athletes at AUB compared to males. Analysis was mainly performed on pre-training measurements for BIVA. 

### 4.3. Future Studies

It is worth extensively exploring the collected data for post-exercise BIA measurements and the different timepoints for urine measurements in a future study. More accurate assessment methods such as blood tests should be used in future studies for the selection of participants based on their complete blood count and lipid profile to determine their health status. Further studies should adjust for possible confounders, increase the female sample size, and investigate the validity of BIVA for hydration assessment in athletes with a specific focus on Xc and Z changes post-exercise.

## 5. Conclusions

In conclusion, there is a significant agreement between BIVA and USG. The sensitivity of classic BIVA in detecting minor changes in hydration status is confirmed graphically and statistically which paves the way towards the validation of BIVA for hydration assessment in athletes. Moreover, the present study demonstrates that male athletes strongly exhibit a specific BIA vector distribution in comparison with a healthy reference non-athletic population of similar age, and compared to female athletes. Further research is needed to develop new specific guidelines for hydration needs before, during, and after training based on bioelectrical changes in the body. Athletes were not extremely hydrated in the present study; hence, these results will be a guiding step for reviewing guidelines to optimize hydration status among AUB varsity team players and strength athletes for better performance. Validating BIVA may be an essential step to allow its use pre- and post- training among university students to assess dehydration in a non-invasive, practical, and inexpensive way.

## Figures and Tables

**Table 1:** Spearman’s correlation for urine color (UC PRE) and raw bioelectrical values Xc, R, and Z, each noted as average PRE in the athletic population.

Correlations						
			Urine Color PRE	Average Xc PRE	Average R PRE	Average Z PRE
Spearman’s rho	Urine color PRE	Correlation Coefficient	1.000	0.012	0.058	0.057
		Sig. (2-tailed)	.	0.888	0.494	0.498
		N	142	142	142	142
	Average Xc PRE	Correlation Coefficient	0.012	1.000	0.746 **	0.749 **
		Sig. (2-tailed)	0.888	.	0.000	0.000
		N	142	148	148	148
	Average R PRE	Correlation Coefficient	0.058	0.746 **	1.000	1.000 **
		Sig. (2-tailed)	0.494	0.000	.	0.000
		N	142	148	148	148
	Average Z PRE	Correlation Coefficient	0.057	0.749 **	1.000 **	1.000
		Sig. (2-tailed)	0.498	0.000	0.000	.
		N	142	148	148	148

** Correlation is significant at the 0.01 level (2-tailed).

**Table 2:** Correlation between BIVA and other methods.

(**a**) Canonical correlation analysis coefficients					
Canonical Function	BIVA Variables	Coefficient	Other methods		Coefficient
Function 1	PA	0.7	Urine Color		0.4
	Xc	0.4	Sweat Rate		−2.3
	R	5.8	USG		−36.3
	Z	−5.8	Body Mass Loss		0.4
Function 2	PA	7.1	Urine Color		0.3
	Xc	−0.5	Sweat Rate		1.4
	R	6.7	USG		62.3
	Z	−6.6	Body Mass Loss		−1.8
Function 3	PA	−6.6	Urine Color		0.4
	Xc	−2.0	Sweat Rate		0.3
	R	−27.1	USG		21.0
	Z	27.2	Body Mass Loss		1.1
Function 4	PA	1.1	Urine Color		−0.2
	Xc	−0.9	Sweat Rate		−1.9
	R	−8.7	USG		83.0
	Z	8.7	Body Mass Loss		0.8
(**b**) Correlation table for BIVA observed and BIVA dimensions					
BIVA Variables	Dimension 1	Dimension 2	Dimension 3		Dimension 4
PA	−0.9	−0.1	−0.3		0.3
XC	0.5	−0.4	−0.2		0.7
R	0.9	−0.1	0.1		0.3
Z	0.9	−0.1	0.1		0.3
(**c**) Correlation table for other methods observed and BIVA dimensions (cross-loadings).					
Other Methods	Dimension 1	Dimension 2	Dimension 3		Dimension 4
Urine Color	0.2	0.1	0.1		−0.0
Sweat Rate	−0.3	−0.0	0.1		−0.0
USG	−0.1	0.1	0.0		0.0
Body Mass Loss	−0.1	−0.1	0.1		0.0
(**d**) Correlation table for BIVA observed and other methods dimensions (cross-loadings).					
BIVA Variables	Dimension 1	Dimension 2	Dimension 3		Dimension 4
PA	−0.4	−0.0	−0.0		0.0
XC	0.2	−0.1	−0.0		0.0
R	0.4	−0.0	0.0		0.0
Z	0.4	−0.0	0.0		0.0
(**e**) Correlation table for other methods and other methods dimensions.					
Other Methods	Dimension 1	Dimension 2	Dimension 3		Dimension 4
Urine Color	0.6	0.4	0.7		−0.3
Sweat Rate	−0.8	−0.1	0.5		−0.4
USG	−0.3	0.5	0.3		0.8
Body Mass Loss	−0.4	−0.6	0.7		0.1
(**f**) Statistical test results for canonical dimensions.					
Canonical Dimensions	Hotelling–Lawley Trace	F-Approximation Statistic	Approximate df1	Approximate df2	*p*-Value
1 to 4	0.2470	2.0766	16	538	0.0082
2 to 4	0.0421	0.6381	9	546	0.7647
3 to 4	0.0166	0.5745	4	554	0.6812
4 to 4	0.0005	0.0655	1	562	0.7981

**Table 3:** Mean, median, and standard deviation of USG in the morning (USG_M), before training (USG_PRE), and after training (USG_POST) for strength males (sport 1), endurance males (sport 2), strength females (sport 1), and endurance females (sport 2).

Statistics						
Gender	Sport			USG_M	USG_PRE	USG_POST
Male	1	N	Valid	54	53	53
			Missing	0	1	1
		Mean		1.020865	1.017159	1.016575
		Median		1.021100	1.016800	1.016200
		Std. Deviation		0.0078826	0.0082862	0.0091917
	2	N	Valid	36	34	36
			Missing	0	2	0
		Mean		1.019469	1.016596	1.017628
		Median		1.019900	1.015500	1.016300
		Std. Deviation		0.0076917	0.0092746	0.0081198
Female	1	N	Valid	29	27	29
			Missing	1	3	1
		Mean		1.017466	1.014887	1.014226
		Median		1.017400	1.015000	1.011600
		Std. Deviation		0.0088995	0.0085637	0.0097921
	2	N	Valid	28	28	28
			Missing	0	0	0
		Mean		1.019300	1.016948	1.016348
		Median		1.018350	1.018800	1.018350
		Std. Deviation		0.0068451	0.0089406	0.0091557

**Table 4:** Multivariate repeated measures ANOVA performed for USG morning, USG PRE, and USG POST between the four sports groups: strength males, endurance males, strength females, and endurance females.

Multivariate Tests ^a^							
Effect		Value	F	Hypothesis df	Error df	Sig.	Partial Eta Squared
USG	Pillai’s Trace	0.129	2.371 ^b^	2.000	32.000	0.110	0.129
	Wilks’ Lambda	0.871	2.371 ^b^	2.000	32.000	0.110	0.129
	Hotelling’s Trace	0.148	2.371 ^b^	2.000	32.000	0.110	0.129
	Roy’s Largest Root	0.148	2.371 ^b^	2.000	32.000	0.110	0.129

^a^. Design: intercept within subjects design, USG. ^b^. Exact statistic.

**Table 5:** Comparison of raw bioelectrical values (R, Xc, Z, and PA) pre- and post-training in athletes by gender and sport type.

	Delta Value	Delta Value/h	Paired *t*-Test	*p*-Value	Cohen’s d
	Mean ± Error Term				
Gender = 1 (Male)					
R/h	−4.50 ± 1.20	−1.32 ± 0.34	7.38	1.1117 × 10^−12^ ***	0.799
Xc/h	−0.37 ± 0.20	−1.00 ± 0.55	3.53	0.000549 ***	0.46
Z	−4.51 ± 1.21	−1.32 ± 0.34	7.33	1.4247 × 10^−11^ ***	0.7957
PA	0.02 ± 0.02	0.32 ± 0.35	−1.82	0.07 *	0.116
Gender = 2 (Female)					
R/h	−4.84 ± 1.27	−1.57 ± 0.42	7.54	3.995 × 10^−11^ ***	0.437
Xc/h	−0.49 ± 0.22	−1.36 ± 0.62	4.34	3.794 × 10^−5^ ***	0.118
Z	−4.85 ± 1.28	−1.57 ± 0.42	7.51	4.665 × 10^−11^ ***	0.433
PA	0.01 ± 0.03	0.23 ± 0.42	−1.04	0.27	0.202
Sport = 1 (Weightlifting)					
R/h	−3.99 ± 2.40	−0.94 ± 0.57	3.33	0.001527 ***	0.791
Xc/h	−0.17 ± 0.39	−0.45 ± 1.01	0.9	0.37	0.616
Z	−3.98 ± 2.42	−0.94 ± 0.57	3.3	0.001677 ***	0.792
PA	0.03 ± 0.03	0.46 ± 0.63	−1.54	0.13	0.179
Sport = 2 (Endurance)					
R/h	−4.64 ± 1.27	−1.40 ± 0.38	7.25	1.956 × 10^−10^ ***	0.475
Xc/h	−0.62 ± 0.22	−1.70 ± 0.60	5.65	2.202 × 10^−7^ ***	0.0212
Z	−4.67 ± 1.28	−1.39 ± 0.39	7.26	1.896 × 10^−10^ ***	0.468
PA	−0.02 ± 0.02	−0.32 ± 0.40	1.64	0.11	0.541

*: significant difference between Pre and Post, *p*-value < 0.05. ***: strongly significant difference between Pre and Post, *p*-value < 0.01.

**Table 6:** Comparison of mean BIA vector between different groups.

Xc/H + R/H+ Z/H + PA (PRE)	Two-Sample Hotelling’s *T*^2^ Test		Paired One-Sample Hotelling’s *T*-Test
All athletes and reference population	*T* = 18.2	*p*-value = 8.79 × 10^−5^ ***	
Male athletes and male reference population	*T* = 18.2	*p*-value = 0.001775 ***	
Female athletes and female reference population	*T* = 4.8	*p*-value = 0.3208	
Strength athletes and general athletic population	*T* = 24.1	*p*-value = 0.000179 ***	
Endurance athletes and general athletic population	*T* = 11.5	*p*-value = 0.02721 ***	
Female endurance athletes and female strength athletes pre–post training			Endurance females: *T* = 17.3; *p* = 0.77
			Strength females: *T* = 11.8; *p* = 0.64
Male endurance athletes and male strength athletes pre–post training			Endurance males: *T* = 22.7; *p* = 0.79
			Strength males: *T* = 43.7; *p* = 0.9

***: strongly significant difference between the two groups, *p*-value < 0.01.

**Table 7:** Mean BIA vector of groups stratified by gender and sport type studied in comparison to the corresponding reference population.

	Welch’s Two-Sample *T*-Test			
Groups	Z/H	R/H	Xc/H	PA
Endurance females vs. reference females	*T* = 94.707 df = 100.05 *p* < 2.2 × 10^−16^ ***	*T* = 1.1654 df = 72.47 *p* = 0.2477	*T* = −0.6267 df = 65.93 *p* = 0.533	*T* = −1.8205 df = 42.21 *p* = 0.0758
	95% CI: [417.46, 435.32]	95% CI: [−7.38, 28.18]	95% CI: [−2.40, 1.25]	95% CI: [−0.45, 0.02]
	$\bar{x}$: 430.55 $\bar{y}$: 4.16	$\bar{x}$: 424.95 $\bar{y}$: 414.55	$\bar{x}$: 39.34 $\bar{y}$: 39.91	$\bar{x}$: 5.31 $\bar{y}$: 5.52
Strength females vs. reference females	*T* = 94.642 df = 100.11 *p* < 2.2 × 10^−16^ ***	*T* = −1.0636 df = 46.66 *p* = 0.293	*T* = −1.9459 df = 55.08 *p* = 0.0568	*T* = −1.0687 df = 43.61 *p* = 0.2911
	95% CI: [417.22, 435.09]	95% CI: [−36.58, 11.28]	95% CI: [−4.02, 0.06]	95% CI: [−0.35, 0.11]
	$\bar{x}$: 430.55 $\bar{y}$: 4.39	$\bar{x}$: 424.94 $\bar{y}$: 437.60	$\bar{x}$: 39.34 $\bar{y}$: 41.32	$\bar{x}$: 5.31 $\bar{y}$: 5.43
Endurance males vs. reference males	*T* = −14.444 df = 76.35 *p* < 2.2 × 10^−16^ ***	*T* = 7.3219 df = 131.25 *p* = 2.198 × 10^−11^ ***	*T* = 1.4205 df = 73.37 *p* = 0.1597	*T* = −8.3279 df = 108.39 *p* = 2.757 × 10^−13^ ***
	95% CI: [−194.14, −147.10]	95% CI: [49.41, 85.98]	95% CI: [−0.57, 3.40]	95% CI: [−1.28, −0.79]
	$\bar{x}$: 376.25 $\bar{y}$: 546.87	$\bar{x}$: 371.39 $\bar{y}$: 303.69	$\bar{x}$: 36.43 $\bar{y}$: 35.01	$\bar{x}$: 5.65 $\bar{y}$: 6.69
Strength males vs. reference males	*T* = 52.851 df = 98.008 *p* < 2.2 × 10^−16^ ***	*T* = 8.9766 df = 148.03 *p* = 1.168 × 10^−15^ ***	*T* = 2.4131 df = 149.38 *p* = 0.01703***	*T* = −9.3513 df = 148.25 *p* < 2.2 × 10^−16^ ***
	95% CI: [359.29, 387.32]	95% CI: [61.40, 96.07]	95% CI: [0.33, 3.35]	95%CI: [−1.35, −0.88]
	$\bar{x}$: 376.25 $\bar{y}$: 2.95	$\bar{x}$: 371.39 $\bar{y}$: 292.65	$\bar{x}$: 36.43 $\bar{y}$: 34.58	$\bar{x}$: 5.65 $\bar{y}$: 6.77

***: statistically significant difference between the two groups, *p*-value < 0.05.

**Table 8:** Reproducibility of BIA device.

**Subject**	**Trial**	**R**	**Xc**	**Z**	**PA**
1	1	747.9	57.1	750.1	4.4
1	2	742.8	56.4	745	4.3
1	3	743.1	55.9	745.2	4.3
**Average**		**744.6**	**56.5**	**746.8**	**4.3**
SD		2.9	0.6	2.9	0.1
**CV (%)**		**0.4**	**1.1**	**0.4**	**1.3**
2.0	1	797.2	67.1	800.1	4.8
2.0	2	790.1	65.4	792.8	4.7
2.0	3	794.8	66.5	797.6	4.8
**Average**		**794.0**	**66.3**	**796.8**	**4.8**
SD		3.6	0.9	3.7	0.1
**CV (%)**		**0.5**	**1.3**	**0.5**	**1.2**
3	1	788.0	75.4	791.7	5.5
3	2	784.5	74.0	788.0	5.4
3	3	786.7	74.1	790.2	5.4
**Average**		**786.4**	**74.5**	**790.0**	**5.4**
SD		1.8	0.8	1.9	0.1
**CV (%)**		**0.2**	**1.0**	**0.2**	**1.1**
4	1	471.3	59.9	475.2	7.2
4	2	466.8	58.6	470.5	7.2
4	3	469.0	58.8	472.7	7.1
**Average**		**469.0**	**59.1**	**472.8**	**7.2**
SD		2.3	0.7	2.4	0.1
**CV (%)**		**0.5**	**1.2**	**0.5**	**0.8**
5	1	503.3	60.2	506.9	6.8
5	2	499.7	60.1	503.4	6.9
5	3	499.5	60.8	503.2	6.9
**Average**		**500.8**	**60.4**	**504.5**	**6.9**
SD		2.1	0.4	2.1	0.1
**CV (%)**		**0.4**	**0.6**	**0.4**	**0.8**
6	1	580.5	62.9	583.9	6.2
6	2	576.7	62.9	580.2	6.2
6	3	574.1	62.7	577.6	6.2
**Average**		**577.1**	**62.8**	**580.6**	**6.2**
SD		3.2	0.1	3.2	0.0
**CV (%)**		**0.6**	**0.2**	**0.5**	**0.0**
**Average**		**645.3**	**63.3**	**648.6**	**5.8**
SD		2.6	0.6	2.7	0.0
**CV (%)**		**0.4**	**0.9**	**0.4**	**0.9**
**ICC**		**0.9**	**0.9**	**0.9**	**0.9**
**TE (%)**		**0.4**	**0.5**	**0.4**	**0.9**

Repeatability of measurements was tested on 6 subjects, 3 males and 3 females, for R, Xc, Z, and PA with an average coefficient of variation (CV) of 0.65%, an average intraclass correlation coefficient (ICC) of 0.99, and an average typical error (TE) of 0.7% indicating reproducibility of measurements, strong consistency, and precision, respectively.

**Table 9:** Anthropometric and bioelectrical parameters for male athletes and reference population.

	Reference Males (*n =* 100)	Endurance Males (*n =* 36)			
Parameter		PRE	POST	Δ-Pre–Post (%)	Δ-Value Ref-Group
**Anthropometric**					
BM (kg)	—	76.7 ± 12.9	76.1 ± 13.0	−0.9 ± 0.8	_
Bioelectrical					
R (Ω)	631.6 ± 91.1	543.1 ± 56.7	535.5 ± 55.9	−1.4 ± 2.3	88.6
Xc (Ω)	62.2 ± 6.5	62.6 ± 8.3	63.4 ± 7.0	2.8 ± 18.7	−0.4
R/h (Ω/m)	304.5 ± 37.2	303.7 ± 32.0	299.4 ± 31.9	−1.4 ± 2.3	0.8
Xc/h (Ω/m)	45.0 ± 4.1	35.0 ± 4.9	35.5 ± 4.2	2.8 ± 18.7	10
PA (°)	5.7 ± 0.8	6.7 ± 0.5	6.8 ± 0.5	1.3 ± 1.9	−1
Z (Ω/m)	634.8 ± 91.0	546.9 ± 56.8	539.3 ± 56.1	−1.3 ± 2.3	87.9
r (R/h, Xc/h)	0.67	0.47	0.75	_—	_—
	**Reference Males (*n* = 100)**	**Strength Males (*n* = 54)**			
**Parameter**		**PRE**	**POST**	**Δ-Pre–Post (%)**	**Δ-Value** **Ref-Group**
**Anthropometric**					
BM (kg)	—	77.8 ± 11.3	77.86 ± 11.3	0.1 ± 0.7	
Bioelectrical					
R (Ω)	631.6 ± 91.1	515.8 ± 53.9	507 ± 50.0	−1.6 ± 1.9	124.6
Xc (Ω)	62.2 ± 6.5	60.9 ± 5.7	59.6 ± 5.4	2.18±5.5	1.3
R/h (Ω/m)	304.5 ± 37.2	292.6 ± 32.8	287.7 ± 30.6	−1.6 ± 1.8	16.8
Xc/h (Ω/m)	45.0 ± 4.1	34.6 ± 3.6	33.8 ± 3.4	−2.0 ± 2.8	11.2
PA (°)	5.7 ± 0.8	6.6 ± 0.4	6.7 ± 0.6	−0.3 ± 1.8	−1
Z (Ω/m)	634.8 ± 91.0	519.5 ± 53.9	510.6 ± 50.1	−1.6 ± 1.8	124.2
r (R/h, Xc/h)	0.67	0.67	0.62	_—	_—

**Table 10:** Anthropometric and bioelectrical parameters for female athletes and reference population.

	Reference Females (*n =* 100)	Endurance Females (*n =* 29)			
Parameter		PRE	POST	Δ-Pre–Post (%)	Δ-Value Ref-Group
**Anthropometric**					
BM (kg)	—	62.2 ± 7.2	62.0 ± 7.3	0.3 ± 1.37	_
Bioelectrical					
R (Ω)	697.8 ± 69.0	680.4 ± 60.1	674.1 ± 59.3	0.93 ± 1.35	17.4
Xc (Ω)	64.6 ± 8.7	65.4 ± 5.6	65.5 ± 6.2	−0.15 ± 9.7	−0.8
R/h (Ω/m)	428.7 ± 45.2	414.6 ± 36.6	410.69 ± 35.7	0.95 ± 0.02	18
Xc/h (Ω/m)	39.7 ± 4.6	39.9 ± 3.9	39.96 ± 4.2	−0.15 ± 7.14	−0.2
PA (Ω)	5.3 ± 0.5	6.7 ± 0.5	6.8 ± 0.5	−1.47 ± 0	−1.4
Z (Ω/m)	700.9 ± 69.1	683.6 ± 59.9	677.4 ± 59.2	0.91 ± 0.02	17.3
r (R/h, Xc/h)	0.60	0.39	0.27	_—	_—
	**Reference Females (*n* = 100)**	**Strength Females (*n* = 29)**			
**Parameter**		**PRE**	**POST**	**Δ-Pre–Post (%)**	**Δ-Value** **Ref-Group**
**Anthropometric**					
BM (kg)	—	57.9 ± 8.1	57.9 ± 8.1	0	
Bioelectrical					
R (Ω)	697.8 ± 69.0	705.0 ± 85.5	698.3 ± 87.9	0.95 ± 2.73	−7.2
Xc (Ω)	64.6 ± 8.7	66.5 ± 6.1	65.8 ± 6.9	1.06 ± 11.6	−1.9
R/h (Ω/m)	428.7 ± 45.2	437.6 ± 56.0	433.5 ± 57.7	0.95 ± 2.95	−8.9
Xc/h (Ω/m)	39.7 ± 4.6	41.3 ± 4.6	40.9 ± 5.1	0.98 ± 0.09	−1.6
PA (Ω)	5.3 ± 0.5	5.4 ± 0.5	5.4 ± 0.6	0	−0.1
Z (Ω/m)	700.9 ± 69.1	708.2 ± 85.4	701.5 ± 87.9	0.95 ± 2.91	−7.3
r (R/h, Xc/h)	0.60	0.63	0.69	_—	_—

Values are the mean ± standard deviation; BM, body mass; R, resistance; Xc, reactance; h, height; PA, phase angle; Z, impedance vector module; r, Pearson correlation coefficient between R/h and Xc/h; %Δ, percent differences Pre to Post; CI, 95% confidence interval.

## Data Availability

The data presented in this study are available on request from the corresponding author. The data are not publicly available due to privacy reasons.

## Supplementary Materials

The following supporting information can be downloaded at: https://www.mdpi.com/article/10.3390/s24186024/s1. Figure S1: Box and whisker plots for phase angle (PA) and urine color (UC) before training in the athletic population. Figure S2: Box and whisker plots for bioelectrical raw value impedance Z and urine color (UC) before training in the athletic population. Figure S3: Box and whisker plots for bioelectrical raw value resistance (R) and urine color (UC) before training in the athletic population. Figure S4: Box and whisker plots for bioelectrical raw value reactance (Xc) and urine color (UC) before training in the athletic population.

## Appendix C. Eligibility Questionnaire

1. Are you a healthy adult between the ages of 18 and 35 years?

▢ Yes
▢ No

2. Do you have any medical problems/illnesses/allergies?

▢ Yes
▢ No

If yes, please specify: ____________________________________

DISEASE	Please tick (✓) as appropriate	Duration of disease (years)	Do you still suffer from any of these conditions? (YES/NO)	Are you following any treatment? (YES/NO)
Diabetes				
Heart Disease				
Hypertension				
High Blood Cholesterol				
Thyroid Disease				
Sleep Apnea				
Other (Asthma, Claustrophobia)				
Cancer				
Other Health Problems				

3. Have you ever been diagnosed with any of the following diseases or do you have any family history?
  Surgical History: ____________________________________________
4. Do you associate any digestive symptoms with eating certain foods?

▢ Yes
▢ No

If yes, please explain: ___________________________________________________

5. Are you part of AUB’s varsity teams?

▢ Yes
▢ No

6. Do you have any injury?

▢ Yes
▢ No
  If yes, please specify: _____________

7. How often do you train?

▢ Daily
▢ Three times per week
▢ Less than three times per week
▢ More than three times per week, please specify: _______________
  For females only:

8. When was the last time you got your period?
  _______________________________________________
9. When do you expect to get it again?
  _______________________________________________
10. What is your normal menstruating cycle? (in days) (In other words, on average how often do you have your period?)
  _______________________________________________
11. Are you on contraceptives or drugs for menstrual cycle treatment?
  ____________________________________________
12. Are you pregnant or planning to become pregnant?
  ____________________________________________

## Appendix D. Pre-Hydration Assessment Questionnaire

  First Name: ________

  Middle Name: _______

  Last Name: ______

  Gender: _______

  Date: ____

  Age: ____

  Telephone number: _______________________

  Email address: ___________________________

  Sport: ___________________________

1. How many training sessions do you have per day on average?
  ▢ One
  ▢ Two
  ▢ Thre
  ▢ Four and abov
  Other, please specify: _____________
2. How much time do you usually spend doing vigorous physical activities?
  _____ hours per day       _____ minutes per day
  _____Don’t know/Not sure
3. What type(s) of physical activity do you engage in? (you can choose more than one):
  ▢ Aerobics/Cardio
  ▢ Anaerobic/Weight Resistance
  ▢ Yoga and Stretching
  Medical/Biochemical/Clinical Assessment:
  How often do you have a bowel movement? _____________________________
  If you take laxatives, what type/brand and how often? _____________________
  Heartburn, Bloating, Gas, Constipation, Diarrhea, Nausea, Vomiting:
  When was the last time you had Blood Test? ____________
  Recent Blood test results:
  Water Retention: Yes No
  Dietary Assessment:
4. Have you been restricting your caloric intake during the past few days?
  ▢ Yes
  ▢ No
  If yes, what is the reason?
5. Are you taking any drugs or medications? If yes, specify name, quantity and duration:______________________________________________________
  ▢ Yes, please specify: _______________
  ▢ No
  ▢ Don’t know
6. Do you consume any of the following foods?
  ▢ Beets
  ▢ Blackberries
  ▢ Carrots
  ▢ Fava beans
  ▢ Rhubarb
7. Which meals do you eat regularly, check all that apply:
  ▢ Breakfast
  ▢ AM Snack
  ▢ Lunch
  ▢ Afternoon Snack
  ▢ Dinner
  ▢ Late Night Snack
8. Are you vegetarian?
  ▢ Yes
  No
9. Have you ever followed a dietary regime?
  ▢ Yes, now
  ▢ Yes, previously
  ▢ No
  If yes, please give details: ____________________________________________________________________________________________________________________
10. Do you take supplements?
  ▢ Yes
  ▢ No
  List the supplements that you take:
  Supplement Type and Brand	How much? When? (Before or after Training)	Reason for Consumption
  		▢ Medical need/deficiency ▢ Due to an inadequate diet ▢ Support immune system ▢ To provide energy ▢ Increase strength/power ▢ To aid recovery ▢ Because everyone else does ▢ Because I am told to ▢ Other (Please state)
11. How many fruits and vegetables in all do you consume daily on average?
  ____________ Fruits _________Vegetables
12. How often do you consume fast foods?
  ▢ Never
  ▢ Two to three times monthly
  ▢ One or two times weekly
  ▢ Daily
13. How many liters of water do you consume daily?
  ▢ Less than one liter
  ▢ One liter to two liters
  ▢ Two to three liters
  ▢ More than three liters
14. How many cups/cans/glasses of tea, coffee, Cola/Pepsi, Red Bull
  (i.e., caffeine containing beverages) do you consume daily? (Please match the columns below accordingly)
  Tea	None to occasionally
  Coffee	One to four
  Cola/Pepsi	Four to ten
  Redbull	More than ten
15. Do you consume alcohol? If yes, specify ________________
  ▢ Never or rarely
  ▢ Only in weekends
  ▢ Once or twice weekly
  ▢ Once or twice daily
  ▢ More than twice daily
16. Do you smoke?
  ▢ Yes
  ▢ No
  If yes, how much on average? __________________________
17. On average how many hours do you sleep per night?
  ▢ More than 10 h
  ▢ Between 8–10 h
  ▢ Between 6–8 h
  ▢ Between 4–6 h
  ▢ Less than 4 h
18. How important do you think good nutrition is to sports performance?
  ▢ Very important
  ▢ Important
  ▢ Moderately important
  ▢ Of little importance
  ▢ Unimportant
19. How important do you think hydration status is to sports performance?
  ▢ Very important
  ▢ Important
  ▢ Moderately important
  ▢ Of little importance
  ▢ Unimportant
20. “The more supplements I take, the better I will perform”:
  ▢ Agree
  ▢ Disagree
  ▢ Strongly disagree
  ▢ Neither agree nor disagree
21. Have you received any previous nutritional advice?
  ▢ Yes
  ▢ No
22. Do you have access to a sports nutritionist/dietitian?
  ▢ Yes, through CHDC
  ▢ Yes, outside AUB
  ▢ No
  Anthropometric Assessment:
  Usual body weight: _________
23. During these past 6 months, did your weight change?
  ▢ Yes, I gained weight
  ▢ Yes, I lost weight
  ▢ No
  ▢ Not sure
  How many kg(s)_____________________?
24. Would you like to receive the hydration tests and body composition results by phone/mail?
  ▢ Yes, by phone
  ▢ Yes, by mail
  ▢ No, I don’t want to receive the results
  Instructions to prepare you for the hydration tests:
  - Avoid caffeine and alcohol consumption prior to the experiment.
  - Do not exercise intensely at least 12 h prior to the experiment.
  - Avoid food in the 4 h prior to the experiment.

## Appendix E. Data Collection Sheet

First Name: ____________________

Middle Name: ___________________

Last Name: ____________________ Date: ____________________

Time: ____________________

E-mail address: __________________________________

Participant #: _______________________

Phone Number (Optional): _______________________

Total training duration: __________________

Gender	
Date of birth/Age	
Sport type	
Eligibility questionnaire	
Pre-hydration assessment questionnaire	
Empty urine cup 1 weight	
Empty urine cup 2 weight	
Empty urine cup 3 weight	
Urine collection 1	
Urine collection 2	
Urine collection 3	
Height	
Body weight 1	
Body weight 2	
Water bottle weight A	
Water bottle weight B	
BIA test 1	
BIA test 2	
BIA test 3	
BIA test 4	

Body weight 1: before training; Body weight 2: after training; Water bottle weight A: before training; Water bottle weight B: after training; BIA test 1: before training; BIA test 2: before training; BIA test 3: after training; BIA test 4: after training.
