# Supplementary material for: Bioelectrical Impedance Vector Analysis (BIVA) for Assessment of Hydration Status: A Comparison between Endurance and Strength University Athletes
Source: Sensors (Basel). 2024 Sep 18;24(18):6024. doi: 10.3390/s24186024 (PMC11435641; doi:10.3390/s24186024)
Supplement: Supplementary file 1 [file sensors-24-06024-s001.zip › sensors-3147953-supplementary.pdf]

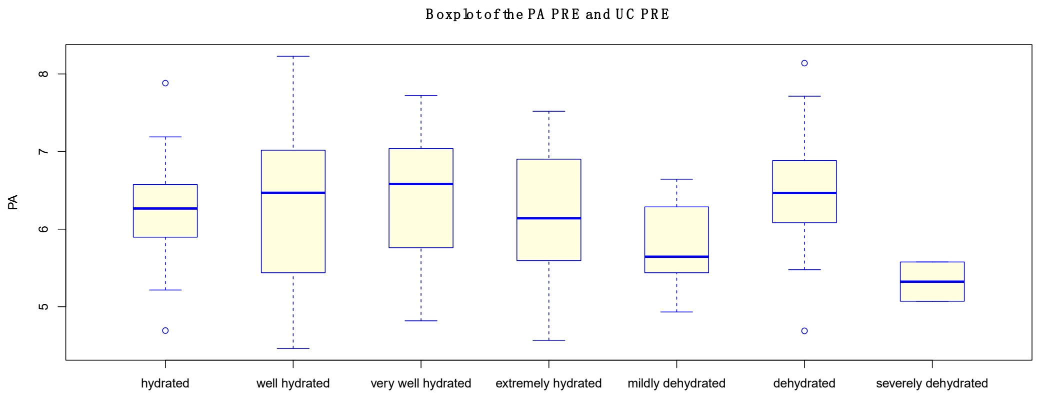

Figure S1: Box and whisker plots for phase angle (PA) and urine color (UC) before training in the athletic population.

The x-axis of the boxplot represents the variable urine color (UC) and the y-axis represents the phase angle (PA). The urine color is divided into seven categories: hydrated, well hydrated, very well hydrated, extremely hydrated, mildly dehydrated, dehydrated, and severely dehydrated. The hydrated group has a median PA of 6.3 with two outliers: one exceeding the upper limit of PA value 7.3 and the other below the lower limit of 5.2. Negative skewness is noted in the hydrated group, indicating that most values were below the median of 6.3.

The well hydrated group has a median PA of 6.6 and it is negatively skewed with an upper band of 8.3 and a lower band below 5. The very well hydrated group has a median PA of 6.8 and it is negatively skewed with an upper band of 7.8 and a lower band of 4.8. The extremely hydrated group has a median PA of 6.1 and it is negatively skewed with an upper band of 7.4 and a lower band below 5. The mildly dehydrated group has a median of 5.3 and it is positively skewed with an upper band of 6.5 and a lower band of 5. The dehydrated group has a median of 6.5 and it has no skewness with an upper band of 7.8 and a lower band of 5.4. The severely dehydrated group has a median of 5.2 and it has no skewness. There is great variability in the median PA between different urine color groups, with the greatest PA observed in the well hydrated group followed by the very well hydrated and extremely hydrated groups, respectively. However, the well hydrated and dehydrated groups have very close median PA values of 6.6 and 6.5.

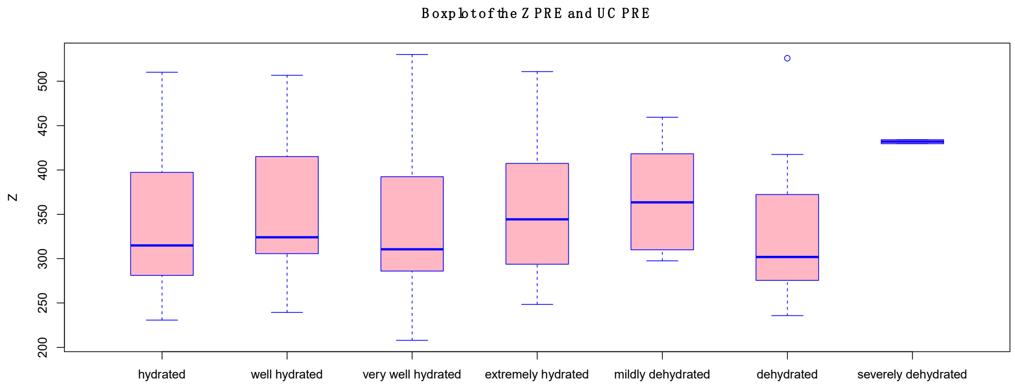

Figure S2: Box and whisker plots for bioelectrical raw value impedance Z and urine color (UC) before training in the athletic population.

The x-axis of the boxplot represents the variable urine color (UC) and the y-axis represents the impedance (Z). The hydrated group has a median Z of 315 (Ω/m). It has an upper limit of 510 (Ω/m) and a lower limit of 225 (Ω/m) and it is positively skewed. The well hydrated group has a median Z of 325 (Ω/m). It has an upper limit of 505 (Ω/m) and a lower limit of 240 (Ω/m) and it is positively skewed. The very well hydrated group has a median Z of 300 (Ω/m). It has an upper limit exceeding 500 (Ω/m) and a lower limit of 210 (Ω/m) and it is positively skewed. The extremely hydrated group has a median Z of 340 (Ω/m). It has an upper limit of 500 (Ω/m) and a lower limit of 250 (Ω/m) and it is slightly positively skewed. The mildly dehydrated group has a median Z of 320 (Ω/m). It has an upper limit of 450 (Ω/m) and a lower limit of 310 (Ω/m) and has no skewness. The dehydrated group has a median Z of 290 (Ω/m). It has an upper limit of 405 (Ω/m) and a lower limit of 240 (Ω/m) and it is positively skewed. The severely dehydrated group has a median Z of 450 (Ω/m). None of the groups have illustrated outliers. However, the severely dehydrated group has missing bands which might be explained by outliers. There is great variability in the median Z between different urine color groups, with the greatest impedance observed in the very well hydrated followed by the hydrated and extremely hydrated groups, respectively. However, the hydrated, very well hydrated and dehydrated groups have very close median Z values.

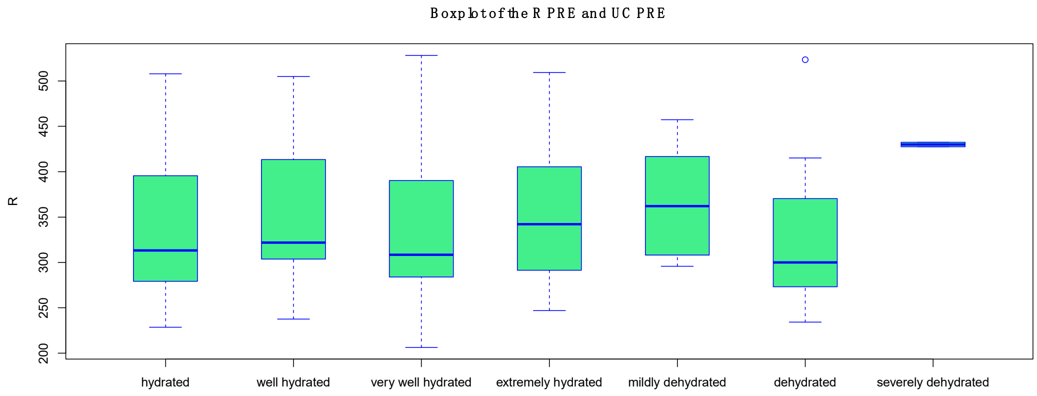

Figure S3: Box and whisker plots for bioelectrical raw value resistance (R) and urine color (UC) before training in the athletic population.

The x-axis of the boxplot represents the variable urine color (UC) and the y-axis represents the resistance (R). The hydrated group has a median R of 315 (Ω). It has an upper limit of 510 (Ω) and a lower limit of 225 (Ω) and it is positively skewed. The well hydrated group has a median R of 320 (Ω). It has an upper limit of 500 (Ω) and a lower limit of 240 (Ω) and it is positively skewed. The very well hydrated group has a median R of 305 (Ω). It has an upper limit exceeding 500 (Ω) and a lower limit of 210 (Ω) and it is positively skewed. The extremely hydrated group has a median R of 350 (Ω). It has an upper limit of 500 (Ω) and a lower limit of 250 (Ω) and it is slightly positively skewed. The mildly dehydrated group has a median R of 350 (Ω). It has an upper limit of 450 (Ω) and a lower limit of 310 (Ω) and has no skewness. The dehydrated group has a median R of 300 (Ω). It has an upper limit of 410 (Ω) and a lower limit of 240 (Ω) and is positively skewed. The severely dehydrated group has a median Z of 440 (Ω). Among all the groups, only the dehydrated group has one outlier exceeding the upper limit. There is great variability in the median R between different urine color groups, with the greatest resistance observed in the very well hydrated group followed by the hydrated group, where it is equal in the extremely hydrated and well hydrated groups, respectively. However, the hydrated and very well hydrated groups have very close median Z values.

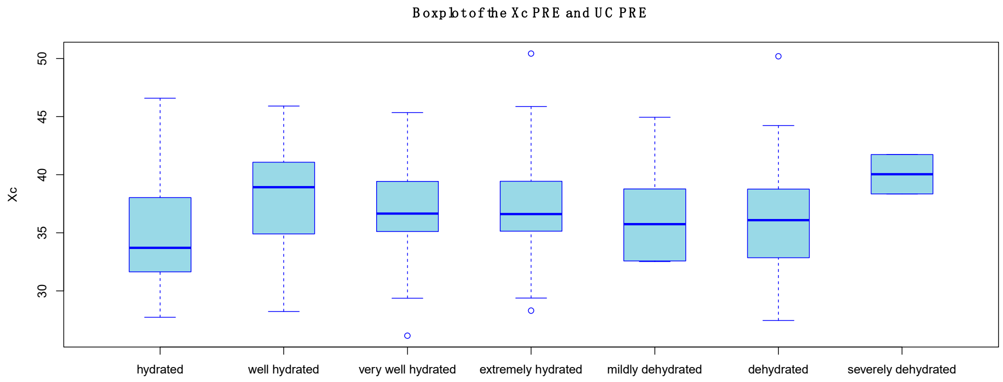

Figure S4: Box and whisker plots for bioelectrical raw value reactance (Xc) and urine color (UC) before training in the athletic population.

The x-axis of the boxplot represents the variable urine color (UC) and the y-axis represents the reactance (Xc). The hydrated group has a median Xc of 34 (Ω). It has an upper limit of 47 (Ω) and a lower limit below 30 (Ω) and it is positively skewed. The well hydrated group has a median Xc of 39 (Ω). It has an upper limit of 46 (Ω) and a lower limit below 30 (Ω) and it is negatively skewed. The very well hydrated group has a median Xc of 37 (Ω). It has an upper limit of 46 (Ω) and a lower limit of 29 (Ω) and it is positively skewed. It has one outlier below the lower limit. The extremely hydrated group has a median Xc of 37 (Ω). It has an upper limit of 46 (Ω) and a lower limit of 29 (Ω) and it is slightly positively skewed. It has two outliers: one above the upper limit and one below the lower limit. The mildly dehydrated group has a median Xc of 35 (Ω). It has an upper limit of 46 (Ω) and a lower limit of 34 (Ω) and is slightly negatively skewed. The dehydrated group has a median Xc of 36 (Ω). It has an upper limit of 43 (Ω) and a lower limit below 30 (Ω) and is negatively skewed. It has one outlier above the upper limit. The severely dehydrated group has a median Z of 40 (Ω) and is evenly distributed with no upper and lower bands. There is great variability in the median Xc between different urine color groups, with the greatest reactance observed in the hydrated followed by the well hydrated and extremely hydrated groups, respectively. However, the very well hydrated, extremely hydrated, and dehydrated groups have similar Xc values.
